# Better, Not Just More—Contrast in Qualitative Aspects of Reward Facilitates Impulse Control in Pigs

**DOI:** 10.3389/fpsyg.2018.02099

**Published:** 2018-11-06

**Authors:** Manuela Zebunke, Maren Kreiser, Nina Melzer, Jan Langbein, Birger Puppe

**Affiliations:** ^1^Institute of Genetics and Biometry, Leibniz Institute for Farm Animal Biology, Dummerstorf, Germany; ^2^Institute of Behavioural Physiology, Leibniz Institute for Farm Animal Biology, Dummerstorf, Germany; ^3^Behavioural Sciences, Faculty of Agricultural and Environmental Sciences, University of Rostock, Rostock, Germany

**Keywords:** impulsivity, delay of gratification, delay choice task, discrimination learning, reward learning, preference test, motivation, pigs

## Abstract

Delay-of-gratification paradigms, such as the famous “Marshmallow Test,” are designed to investigate the complex cognitive concepts of self-control and impulse control in humans and animals. Such tests determine whether a subject will demonstrate impulse control by choosing a large, delayed reward over an immediate, but smaller reward. Documented relationships between impulsive behavior and aggression in humans and animals suggest important implications for farm animal husbandry and welfare, especially in terms of inadequate social behavior, tail biting and maternal behavior. In a preliminary study, we investigated whether the extent of impulse control would differ between quantitatively and qualitatively different aspects of reward in pigs. Twenty female piglets were randomly divided into two groups, with 10 piglets each. After a preference test to determine individual reward preference among six different food items, a discrimination test was conducted to train for successful discrimination between different amounts of reward (one piece vs. four pieces) and different qualitative aspects of reward (highly preferred vs. least preferred food item). Then, an increasing delay (2, 4, 8, 16, 24, 32 s) was introduced for the larger/highly preferred reward. Each piglet could choose to get the smaller/least preferred reward immediately or to wait for the larger/highly preferred reward. Piglets showed clear differences in their preference for food items. Moreover, the “quality group” displayed faster learning in the discrimination test (number of sessions until 90% of the animals completed the discrimination test: “quality group”−3 days vs. “quantity group”−5 days) and reached a higher level of impulse control in the delay-of-gratification test compared to the “quantity group” (maximum delay that was mastered: “quality group”−24 s vs. “quantity group”−8 s). These results demonstrate that impulse control is present in piglets but that the opportunity to get a highly preferred reward is more valued than the opportunity to get more of a given reward. This outcome also underlines the crucial role of motivation in cognitive test paradigms. Further investigations will examine whether impulse control is related to traits that are relevant to animal husbandry and welfare.

## Introduction

During their daily life, animals face many decisions, including social conflict, predator avoidance, feeding and mating. Therefore, time is a critical factor in each choice situation, resulting in an intertemporal choice problem, e.g., fight now and risk injury or withdraw and fight later, or, leave a food patch now after eating a small amount of food, or stay longer to obtain more (Stevens and Stephens, 2010, p. 361 ff.). Studies on intertemporal choice are closely related to studies on self-control or impulse control aimed at investigating the trade-off between far-sighted decisions and short-term temptations (Logue, 1988; Berns et al., 2007). Impulse control as a cognitive function is part of the neural network of inhibitory control, which is in turn a core element of executive functions (Diamond, 2013). Impulse control enables behavior control as well as withstanding internal predispositions and external temptations in order to adopt appropriate behaviors for different situations (Bari and Robbins, 2013). To investigate impulse control, diverse paradigms have been used to address two different forms of impulsivity, impulsive action (motor impulsivity) and impulsive choice/decision-making (cognitive impulsivity; Monterosso and Ainslie, 1999; Winstanley et al., 2006).

The main approach to studying impulsive choice uses tests involving delay of gratification/reward, wherein subjects have the choice between a more immediate but smaller reward and a delayed, larger reward (Leonardi et al., 2012; Beran, 2015). Two different types of tasks, namely, delay choice and delay maintenance, can be used to investigate different components of delayed gratification (Addessi et al., 2013; Paglieri et al., 2013). Delay choice tasks are directly linked to intertemporal choice (see above), giving the subjects the choice between a smaller, sooner reward and a larger, delayed reward with no possibility to change their choice (Stevens and Mühlhoff, 2012). Such intertemporal choice or self-control tasks have been successfully used in a wide range of species, such as insects (e.g., Cheng et al., 2002), fish (e.g., Mühlhoff et al., 2011), birds (e.g., Ainslie, 1974; Vick et al., 2010), rodents (e.g., Tobin and Logue, 1994; Brunner and Hen, 1997), and primates (e.g., Stevens et al., 2005; Rosati et al., 2007). These studies show that in addition to humans, animals can also choose to wait for a larger/better outcome, at least up to a certain delay. With increasing delay, the choice of the larger/better reward usually decreases in the form of a hyperbolic function, which is suggested to be due to devaluation or discounting of the reward (Reynolds et al., 2002; Madden and Bickel, 2010). Thus, delay discounting results in mainly impulsive choices across a wide range of species (Stevens and Stephens, 2010). The same authors point to a critical aspect: “In an evolutionary approach, a preference for immediate rewards appears not impulsive but adaptive in naturally occurring behavioral situations. […] Decision mechanisms adapted to a common foraging problem may not work as well in an artificial laboratory situation.” (Stevens and Stephens, 2010, p. 383). In contrast, in delay maintenance tasks, such as the accumulation task or the exchange task, subjects are able to take a smaller reward at any time or can choose to wait until the delivery of the larger/better reward (Beran, 2002; Evans and Beran, 2007; Beran et al., 2016). Studies on sustaining the choice of a delay to reward in animals, mainly on birds and primates, show that they are able to withstand temptations for up to several minutes in favor of a larger or more preferred reward (Stevens et al., 2011; Evans et al., 2012; Addessi et al., 2013; Auersperg et al., 2013; Hillemann et al., 2014; Koepke et al., 2015).

Many studies have investigated the features and mechanisms of intertemporal choice and delayed gratification in terms of species ecology and the evolution and economy of decision making in humans and animals (e.g., Loewenstein et al., 2003; Stevens, 2014; Beran, 2015). Several decades ago, Walter Mischel performed studies on delay-of-gratification in children, which later became known as the “Marshmallow Test” (Mischel et al., 1989). The children could choose to either take one marshmallow immediately (small reward) or to wait for the return of the experimenter (delay) to get two marshmallows (large reward). Subsequent studies revealed a surprising relationship between the degree of impulse control as a child and academic and social competence as well as stress coping and attention abilities later in life (Mischel et al., 2011). Several studies in humans and animals have also suggested that individual variation in impulsivity is a behavioral or even a personality trait (Kirkpatrick et al., 2014; Velázquez-Sánchez et al., 2014; Ciardelli et al., 2017) that is partly related to aggressive behavior (Brunner and Hen, 1997; Cervantes and Delville, 2007; Coppens et al., 2014) and affected by stressors (Metcalfe and Mischel, 1999). This line of research is valuable to be extended to a group of species, not much investigated so far, for which cognitive research on self-control and possible links to aggression and stress coping behavior would be beneficial in terms of health, welfare and animal protection. In farm animals, many problems still exist on-farm in terms of maternal behavior, injurious behavior (e.g., aggression, tail biting, feather pecking) and stereotypes (Keeling and Jensen, 2017). It can be noted that, despite all the animals in a group have nearly the same environmental conditions, not all animals show these behavioral problems. Thus, individual variation in impulse control seems to be a promising approach to understanding individual variation in stress coping behavior and mechanisms that finally lead to behavioral problems in farm animals, such as pigs.

Pigs, as omnivores, exhibit a flexible foraging ecology and occupy a wide variety of habitats (Leaper et al., 1999). Moreover, their brains show a developed prefrontal cortex comparable to that of humans and non-human primates (Kornum and Knudsen, 2011), providing them with pronounced cognitive abilities (Zebunke et al., 2011; Marino and Colvin, 2015; Düpjan et al., 2017). The prefrontal cortex is also linked to a capacity for impulse control (Fuster, 2015). Thus, pigs have a certain capacity for impulse control, which has been previously explored in only one study (Melotti et al., 2013). In this study, the authors investigated the behavior of pigs in a delay choice paradigm. They found that the pigs were willing to wait for 12 to 50 s for a larger reward. The aim of the present experiment was to expand knowledge and to examine the effect of different contrasts in reward (different amount [quantitative aspect] vs. differentially preferred items [qualitative aspects]) on the level of impulsivity/impulse control in a delay maintenance task in pigs. This is a pilot study for a larger research project investigating the phenomenon of impulse control in pigs and its relationships with personality, social behavior, emotional coping and other cognitive capacities, as well as its possible impact on animal husbandry and welfare.

## Animals, materials, and methods

### Ethical statement

All animal care and experimental procedures were performed in accordance with the German welfare requirements for farm animals and the ASAB/ABS Guidelines for the Use of Animals in Research (Anonymous, 2016). All experimental procedures were approved by the Committee for Animal Use and Care of the Ministry of Agriculture, Environment, and Consumer Protection of the Federal State of Mecklenburg-Vorpommern, Germany (ref. no. 7221.3-2-016/16).

### Subjects and housing

The experiment was conducted between May and July of 2016. We used 20 female German Landrace piglets, born in April of 2016 and raised in scan farrowing pens in the experimental pig unit of the Leibniz Institute for Farm Animal Biology (FBN), Dummerstorf, Germany (Stabenow and Manteuffel, 2002). The piglets were weaned at 28 days of age and transported to an experimental room in the same unit. The piglets were randomly divided into two groups of 10 piglets each and were moved into adjacent pens measuring 3 × 5 m. The pens contained several nipple drinkers with water *ad libitum* and a trough with an animal to feeding space ratio of 2:1. The piglets received 90% of their recommended feeding amount divided into two portions of 45%: once around midday, after the experiment, and the rest at ~3:30 p.m. (Lindermayer et al., 1994). This procedure was chosen to ensure motivation for participation in the experiment due to empty troughs in the morning. The piglets could receive the other 10% of the recommended feeding amount by consuming the available rewards during the experiment. The partially slatted floor was cleaned daily and covered with a mixture of chopped straw, wood shavings and hemp pellets. The pens each contained four or five balls of hard rubber, fixed with metal chains and used as environmental enrichment. At the beginning, directly after weaning, the pens were equipped with heat lamps to help the piglets maintain their body temperature. A few weeks later, with rising outdoor temperatures, the heat lamps were removed. In addition to natural daylight, the experimental room was artificially illuminated from 7:30 a.m. to 4:00 p.m. During the experiment, one piglet in one group was treated due to lameness, and one piglet in the other group was treated due to reddish urine.

### Experimental setup

**Figure 1:**
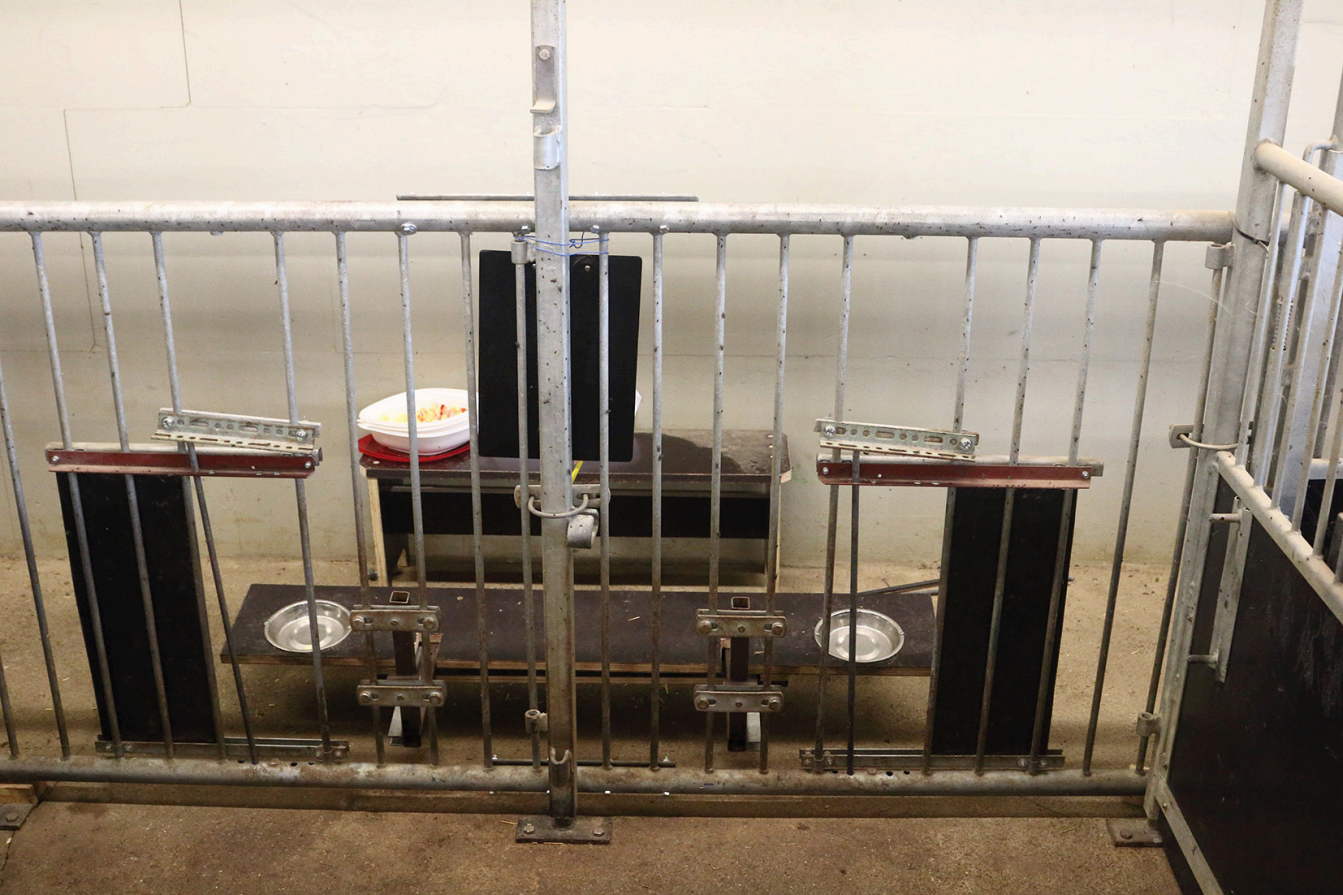
Experimental pen with the experimental setup: grating with closable openings to the reward dishes fixed in a sliding board. The experimenter was positioned behind the grating and managed the openings and the sliding board, as well as baiting the reward dishes.

The experimental pen was located near the holding pens of the piglets so that it was not necessary to transport the piglets a long way and isolate them socially during the individual experiments. The experimental pen was a combination of two single pens and measured 2 × 2 m (Figure 1). Opposite to the entrance, the pen wall was replaced by a metal grid with two openings of ~20 cm each (made by removing one bar in each case). The openings were wide enough that a piglet could put its head and ears through. The width of the openings could be adjusted for the growing piglets by metal bars, inserted separately, that could be fixed at the top to prevent their removal by the piglets. The distance between the openings was ~1 m. A wooden sliding board (1.20 × 0.25 m) with two metal puppy feeding dishes (diameter: 20 cm) inserted at the level of the openings was installed behind the grid to present the rewards [see Figure 1, see also (Nawroth et al., 2015)]. The experimenter was positioned behind the sliding board to manually operate it. Two wooden sliding doors were attached between the grid and the sliding board so that access to the rewards could be regulated. With open sliding doors and a pulled-back sliding board, the piglets could not reach the rewards. Six different food items, differing in qualitative aspects (i.e, visual and olfactory cues), were used during the experiment as potential rewards: standard food pellets, uncooked pasta (Penne), chocolate M&M's®, pieces of fresh apple (Jonagold), pieces of cheese (young Gouda) and chicken sausage.

### Experimental procedure

One experimenter managed the experimental protocol weekdays between 8 and 11:30 a.m. All the piglets were individually marked. The animals of one group (“quantity”) started all the tests first, followed by the animals in the other group (“quality”). Within each group, the order of the piglets was pseudorandomized for each session.

#### Habituation

The experimental procedure started 4 days after weaning with a stepwise habituation of the piglets to the experimental pen, the experimental setup and the food rewards. For this purpose, the experimenter was present, the sliding doors opened, the sliding board was fixed toward the grid and the dishes were filled with standard food pellets (day 1–3) or a mixture of food rewards (day 4–7). As a first step, in two sessions (day 1), five piglets per group could explore the experimental pen for 10 min. This process was followed by two sessions per day (day 2–5), with two piglets exploring the experimental pen for 5 min. The first session of day 6 followed the same procedure (two piglets for 5 min). In the second session, each piglet could individually explore the experimental pen for 2 min, as was the procedure for the two sessions on day 7. After each of the two sessions per day and before regular feeding, a mixture of all the food rewards was placed in the standard feeding troughs of both groups to habituate the piglets to the unknown food and to prevent potential neophobia (Roura et al., 2008). The habituation was followed by a preference test, lasting 3 days. Thereafter, another habituation session followed to refamiliarize the animals with the experimental setup. Then, the function of the sliding doors (initially closed) and the sliding board (initially pulled back) were introduced. Each piglet completed 12 trials with one piece of its most preferred food item as a reward in one dish. In each trial, one of the sliding doors was opened and, when the piglet put its head and ears through the opening (i.e., it made a choice), the sliding board with the reward dish was pushed toward the piglet. After the reward was consumed or after 30 s with no choice made (timeout), the sliding door was closed, and a new trial began. The order of the reward side opened to each piglet was pseudorandomized (6 × left, 6 × right).

#### Preference test

The preference test was adapted from Hillemann et al. (2014) and was also performed in the experimental pen. We used two spoons that were placed close to each other through the metal grid to present two different food items to the piglets (one piece of each). We used the six different food items mentioned above (pellets, penne, M&M's®, apple, cheese, sausage), which resulted in 15 test combinations of two items. Each combination was repeated three times, for a total of 45 trials per animal (a total of 900 trials across all animals). One session, consisting of 15 consecutive trials, was completed per day and per piglet so that all 15 possible food combinations were presented in pseudorandomized order. The food item that was ingested first was considered preferred. In cases where no choice was made by the piglet within 30 s (timeout), the trial outcome was considered an “omission” or refusal of both food items. By analyzing the choices and omissions for each piglet, the most and the least preferred food items were determined. In cases where there was an equal preference for two or more food items (this was the case for three piglets), we decided upon cheese, as this was among the most highly preferred foods and seemed to be a widely desired item. The least preferred reward was pellets and we did not continue to use this reward in further trials, as it was difficult to dose compared to pieces of penne or M&M's®.

#### Discrimination test

During the discrimination test, the piglets learned to discern differences in the reward presented on two specified sides of the sliding board. One group received different amounts of a reward (i.e., contrast in the quantitative aspect, one piece vs. four pieces of the subject's most preferred food item), and the other group received differentially preferred rewards, referring to qualitative aspects of reward (one piece of the most vs. one piece of the least preferred food item). For simplicity, the groups were further referred to as “quantity group” and “quality group.” The rewarded side (left vs. right, larger/highly preferred vs. smaller/least preferred) was always the same for individual piglets and was pseudorandomized across the piglets, with an equal distribution of side-reward property combinations (i.e., 5 × left larger, 5 × left smaller, 5 × left highly preferred, 5 × left least preferred). Each piglet completed one session per day, with 12 trials each, and a 30 s timeout; if the piglet refused to make a choice within 30 s, the trial was rated as “omission.” Each trial started with the sliding board pulled back out of reach for the piglets, and the doors closed. After equipping both reward dishes with the according rewards, the door(s) were opened and the piglet was able to put its head through one of the openings. Each session started with four forced choice trials wherein only one of the two sliding doors was opened to ensure that the piglets experienced the reward opportunities on both sides. First, in two trials, the side with the larger/highly preferred reward was opened, followed by two trials with the side with the smaller/least preferred reward. In the following eight free choice trials, both sliding doors were opened, giving the piglet the opportunity to make a choice. A choice was made when the piglet put its head and ears through the opening. In this case, the table was immediately pushed to the piglet so that it was able to consume the chosen reward. Thereafter, the table was pulled back, both doors closed and a new trial began. The piglets were not able to consume the other, un-chosen reward, as the sliding door on that side was closed immediately after the piglet had made its choice. The discrimination test was performed until each piglet passed the learning criterion, meaning it chose the larger/highly preferred reward in at least seven of the eight free choice trials, which corresponds to a significant preference according to the binomial test (Melotti et al., 2013). When a piglet passed the learning criterion, it started directly in the next session with the delay maintenance test for impulse control.

#### Delay maintenance test

The test procedure in the delay maintenance test was nearly the same as in the discrimination test (see above). Here, the number of forced choice trials was decreased, whereby the number of free choice trials was increased and an increasing delay was introduced to the free choice trials [see (Melotti et al., 2013)]. Thus, in the case of choosing the smaller/least preferred reward, the table with the reward was pushed immediately to the piglet, whereas in the case of choosing the larger/highly preferred reward, the table was pushed to the piglet only after a certain delay, keeping both reward options in view during the delay. Therefore, the piglet always had the opportunity to revise its decision during the delay and to switch to the other side that provided an immediate reward [as examples see Supplementary Videos for waiting (Supplementary Video 1), immediate reward (Supplementary Video 2), and switching (Supplementary Video 3)]. Thus, the current task is seen as a delay maintenance task, despite the fact that the experimental setup is similar to classical intertemporal choice tasks testing for delay choice (Addessi et al., 2014). Again, a 30-s timeout was used: if the piglet refused to make a choice within 30 s, the trial was rated as “omission.”

Each session started with two forced choice trials, first opening the side with the larger/highly preferred reward and after the piglet's choice (i.e., putting head and ears through the opening), giving access to the reward only after a delay, as in the following free choice trials. This process was followed by a one-time opening of the side with the smaller/least preferred reward, giving access without delay. Thereafter, there were ten free choice trials, giving the piglet the opportunity to wait for the larger/highly preferred reward or to choose the immediate but smaller/least preferred reward. The delay increased in the following steps: 2 s, 4 s, 8 s, 16 s, 24 s, 32 s (40, 50 s–these steps were planned for but not tested). Similar to the discrimination test, we used a significant binomial test to decide whether an animal significantly chose to wait for the larger/highly preferred reward and thus successfully passed a delay step, meaning the piglet waited for the larger/highly preferred reward in at least nine out of 10 free choice trials. Consequently, the animal received the next higher delay in the following session. We also used the binomial test to decide whether an animal significantly failed a delay step due to choosing the immediate reward, switching from waiting to the immediate reward during the delay or omitting a choice until timeout (for at least nine out of the 10 free choice trials). Consequently, for these animals the experiment was finished. Animals that showed indifferent behavior (i.e., no clear waiting and no clear failing) received the same delay again in the next session, i.e. the animals repeated the same delay step as long as they did not reach the criteria for successful waiting (next delay step in the following session) or significant failing (experiment finished), respectively. The experiment was finished when all the animals left the delay maintenance test due to significantly failing a delay step.

### Statistical analysis

In each trial of the preference test, the animal could make a choice by choosing one food item over another for ingestion, or, could omit a choice by refusing both food items. We first calculated the total proportion of choices and omissions with regard to the number of total trials. The proportions of choices and omissions of the single food items are presented for both groups as well as for each individual.

For the discrimination test the maximum number of sessions needed to pass the learning criterion was compared between the groups using the Mann-Whitney test (due to the non-normal distribution of the data).

In the delay maintenance test, we excluded subjects from the experiment as soon as they significantly failed to wait. Therefore, with increasing delay, we had to deal with a decreasing number of animals. Nevertheless, to statistically compare both groups, we first examined the total number of sessions completed by the two groups using an unpaired *t*-test (after confirming normal distribution of the data). Then, we analyzed the maximum delay step that was successfully mastered by each individual in both groups. Therefore, we transformed the categorical delay steps (2, 4, 8, 16, 24, 32 s) into a continuous variable (delay step: 1, 2, 3, 4, 5, 6) and used the Mann-Whitney Test (due to non-normal distribution of the data) to test the median scores of the two samples for significant differences. We also categorized the piglets' strategies as wait (for the larger/highly preferred reward), immediate (choosing the smaller/least preferred reward), switch (revision of the choice from waiting to immediate reward during the delay) and omit (making no choice within 30 s) and calculated the proportion of trials for each strategy. The single strategies were compared between the groups across all the trials using an unpaired *t*-test (after confirming normal distribution of the data) and, when necessary, a Welch correction due to unequal standard deviations (this was the case for the switching strategy). For one of the piglets from the quality group, the delay step of 4 s was accidently skipped, so that those data are missing (see Supplementary Table 4).

All the statistical analyses were performed in GraphPad InStat (Version 3.06, GraphPad Software, La Jolla California USA, www.graphpad.com).

## Results

### Preference test

**Figure 2:**
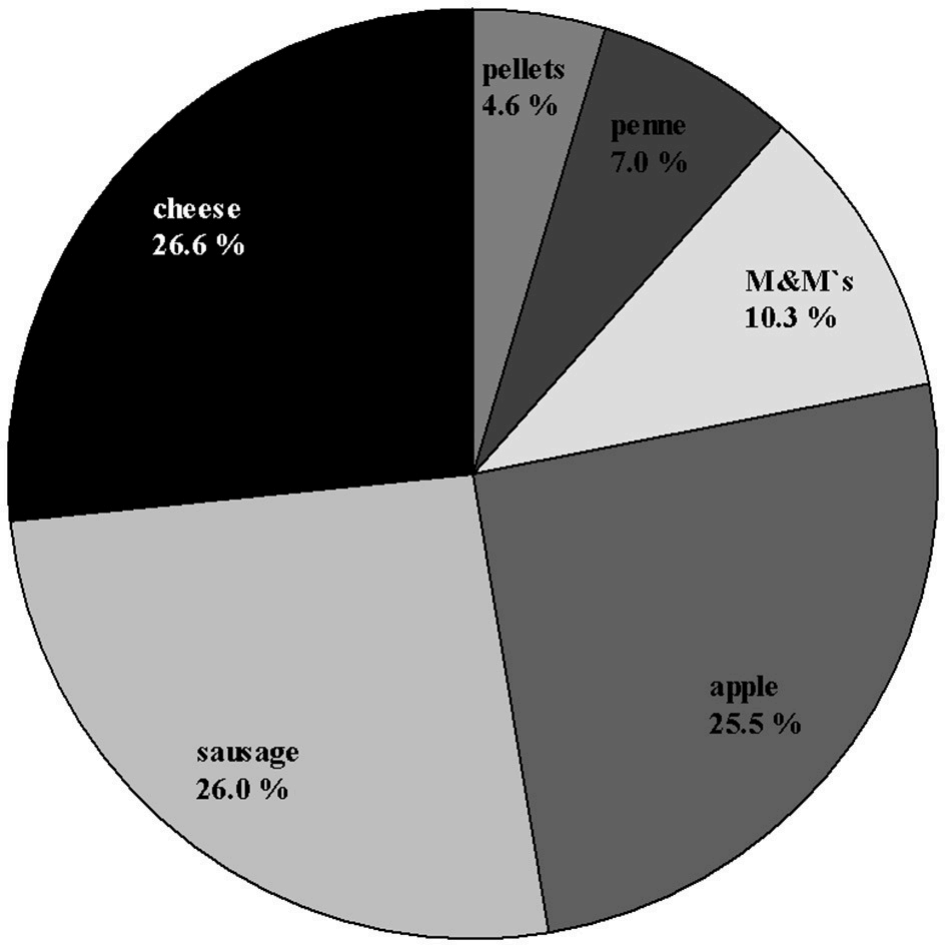
Percentage distribution of choices (*N* = 804) across the rewards used in the preference test for all animals in both groups.

Out of 900 total trials, the piglets made 804 choices (89.3%) and 96 omissions (10.7%, see Supplementary Table 1). The most preferred items were apple, cheese and sausage. These items accounted for more than 75% of the preferences (see Figure 2; Supplementary Table 2). In total, 17 animals preferred one item more than all the others, while three animals preferred two or three items equally. This outcome resulted in 24 highly preferred items (rather than 20–the number of animals): 7 × sausage, 7 × cheese, 6 × apple, 3 × M&M's®, 1 × penne. Roughly summarized, the animals preferred 14 × savory tastes, 9 × sweet taste, and 1 × neutral taste. The most refused items were pellets, penne and M&M's®, especially in the three combinations of these items.

### Discrimination test

**Figure 3:**
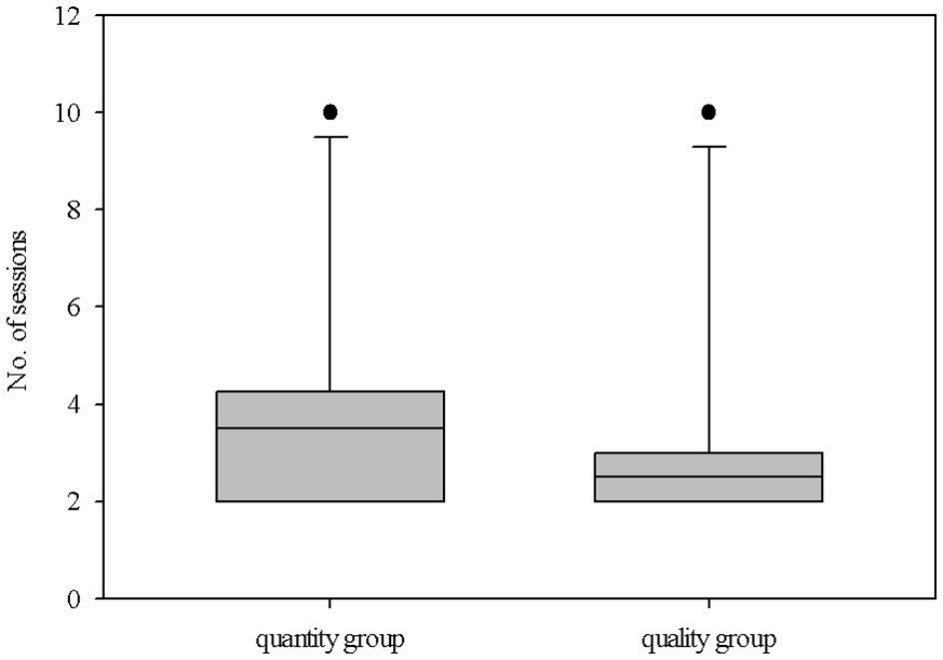
Performance of the animals in both groups (quantitative difference in reward [amount: 1:4] vs. qualitative difference in reward [differentially preferred items: low: high]) during the discrimination test, i.e., the number of sessions needed to reach the learning criterion (significantly choosing the larger/highly preferred reward). The boxplot shows the distribution of the data from both groups with the 25, 50th (median), and 75th percentiles as gray boxes, the 90th percentile as a whisker and black circles as outliers.

The individual number of sessions needed to pass the learning criterion varied: it ranged from 2 to 10 sessions. In both groups, one piglet needed 10 sessions. Apart from that, the other nine piglets of the “quantity group” needed a maximum of five sessions to pass the learning criterion, while the other nine piglets of the “quality group” needed a maximum of just three sessions. On average, there was no significant difference between the groups (“quantity group”: 3.9 ± 2.4 sessions, “quality group” 3.2 ± 2.4 sessions; *U* = 33.0, *P* = 0.2, n1 = n2 = 10; Figure 3). The proportion of omissions was quite low. During a total of 568 free choice trials, only 22 omissions occurred (3.9%) in just two animals (“quantity group”: animal 5, 4 omissions; “quality group”: animal 8, 18 omissions). An overview of the individual choices (larger/highly preferred, smaller/least preferred, omission) is given in Supplementary Table 3.

### Delay maintenance test

**Figure 4:**
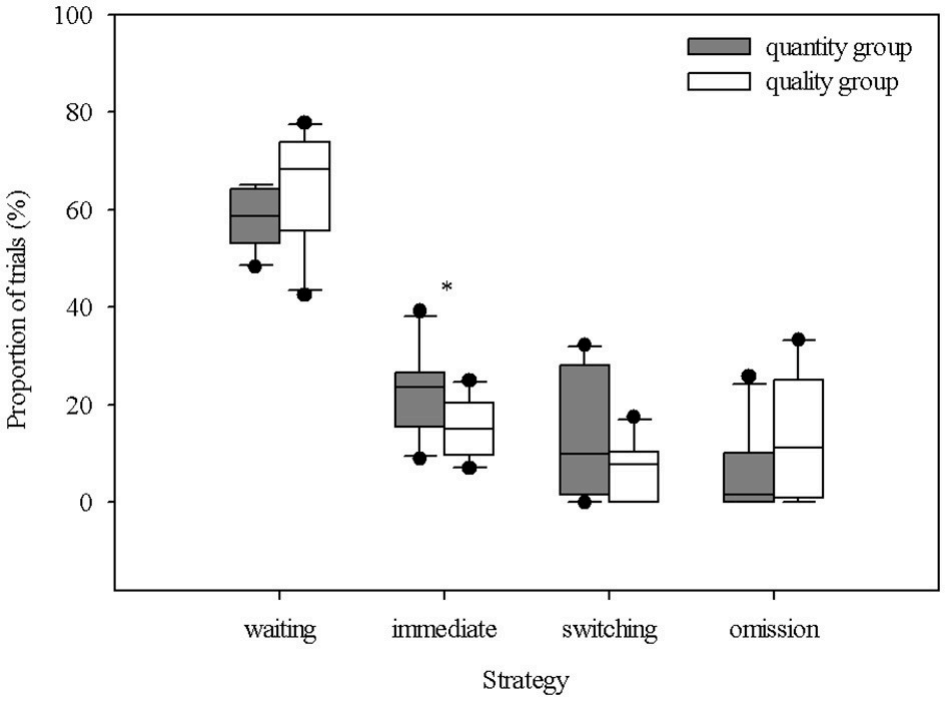
Percentage distribution of the different behavioral strategies during decision-making across all trials of the animals in both groups (quantitative difference in reward [amount: 1:4] vs. qualitative difference in reward [differentially preferred items: low: high]) during the delay maintenance test. The boxplot shows the distribution of the data within the single strategies with the 25, 50th (median), and 75th percentiles as gray boxes, the 90th percentile as a whisker and black circles as outliers. The asterisk indicates a significant difference between both groups revealed with an unpaired *t*-test (* = *P* < 0.05).

The total number of sessions completed by the individual piglets ranged from three to 13, and the total number of trials across all the piglets was 1650. In comparing the total number of sessions between the groups, it emerged that the “quantity group” completed on average more sessions than the “quality group,” but the difference was not significant (“quantity group”: 9.2 ± 3.1 sessions, “quality group”: 7.3 ± 2.8 sessions; *t*_18_ = 1.435, *P* = 0.168). The delay steps that were successfully passed by the piglets ranged from 0 s (that is, the piglets did not even master the first delay step of waiting 2 s for the reward) to 24 s. The maximum delay step, mastered by two animals from the “quantity group,” was 8 s, while two animals from the “quality group” mastered the maximum of 24 s. Comparing the maximum delay steps mastered between both groups showed that the “quantity group” successfully mastered, on average, the second delay step of 4 s (2.0 ± 0.67) and the “quality group” showed a tendency to successfully master the next higher delay step of 8 s (3.0 ± 1.83, *U* = 27.0, *P* = 0.088, n1 = n2 = 10). Table 1 illustrates the decreasing number of animals and the decreasing proportion of trials with waiting with each increasing delay. The proportions of trials with choosing the immediate reward, switching and omissions did not show a clear pattern across the increasing delay steps. The proportions of the different strategies shown by the piglets in relation to all the trials are shown in Figure 4. The “quantity group” chose the immediate reward in a significantly larger proportion of trials than did the “quality group” (“quantity group”: 22.8 ± 8.3%, “quality group”: 15.0 ± 6.1%; *t*_18_ = 2.392, *P* = 0.028). The proportion of trials with successful waiting (“quantity group”: 58.2 ± 6.3%, “quality group”; 64.5 ± 11.3%; *t*_18_ = 1.525, *P* = 0.145), switching (“quantity group”: 13.2 ± 12.3%, “quality group”: 7.2 ± 5.8%; *t*_18_ = 1.41, *P* = 0.176) and omissions (“quantity group”: 5.8 ± 8.3%, “quality group”: 13.4 ± 12.4%; *t*_18_ = 1.622, *P* = 0.122) was not significantly different between groups. Nevertheless, numerical differences indicate that animals of the “quantity group” were prone to show the switching strategy, whereas animals of the “quality group” were prone to omit any choice. However, the high standard deviations indicate a high individual variation. An overview of the individual choices (successful waiting, switching, immediate reward, omission) is given in Supplementary Table 4.

**Table 1:** Overall performance of the animals in both groups (quantitative difference in reward [amount] vs. qualitative difference in reward [differentially preferred items]) during the delay maintenance test.

**Performance/group**	**Delay step**											
	**2 s**		**4 s**		**8 s**		**16 s**		**24 s**		**32 s**	
	**Quantity**	**Quality**	**Quantity**	**Quality**	**Quantity**	**Quality**	**Quantity**	**Quality**	**Quantity**	**Quality**	**Quantity**	**Quality**
Successful animals [*n*]	10	8	8	7	2	7	0	5	0	2	0	0
Waiting [% of trials]	73.21	74.21	67.74	87.78	40.00	77.78	22.86	53.85	0	48.57	0	25.71
Immediate [% of trials]	17.86	12.63	19.35	10.00	36.54	10.56	24.29	20.77	0	15.71	0	17.14
Switching [% of trials]	4.64	0	2.26	0	18.46	1.67	42.86	23.08	0	15.71	0	20.00
Omission [% of trials]	4.29	13.16	10.65	2.22	5.00	10.00	10.00	2.31	0	20.00	0	37.14

## Discussion

The piglets in the current study showed individual food preferences. While we found no influence of the type of contrast between rewards (amount vs. differentially preferred items) on learning in a discrimination test, the level of impulse control varied depending on the contrast between rewards.

### Preference test

The individual preferences for potential food reward items and the distribution of choices made across the food items were quite heterogeneous and diverse (Figure 2). Preferences ranged from a near equal distribution of choices across all the food items to a sharply skewed distribution for individuals who refused half of the food items completely. It is obvious that in cognitive experimental setups, where motivation is crucial, the reward type itself plays an important role. In many experiments, the same food reward for all animals is used to standardize the experiment. In the case of pigs, either standard food was used after food deprivation (Dantzer et al., 1980; de Jong et al., 2000; Elizabeth Bolhuis et al., 2004), or mostly sweet food rewards were used, such as M&M's® (de Jonge et al., 2008; Gieling et al., 2013), apple/applesauce (Douglas et al., 2012; Melotti et al., 2013; Düpjan et al., 2017), raisins (McLeman et al., 2005; de Jonge et al., 2008) and chocolate raisins (Bolhuis et al., 2013). In the current study, in more than half of the choices made, a food item with a savory taste was preferred (cheese, sausage), and in a another third, a food item with a sweet taste was preferred (apple, M&M's®). The results clearly demonstrate that in pigs, similar to humans, tastes are quite different; consequently, the motivation to do something to get a standardized food reward differs among individuals. Therefore, in experiments using a food reward, it is more appropriate to perform a preference test in advance and to use individually preferred food items in order to attain high motivation. However, this approach must be tested in further experiments, to determine whether a preference for a special food item, once developed, is stable across time in pigs.

### Discrimination test

The piglets needed 2–10 sessions (16 to 80 free choice trials) to reach the learning criterion (significantly choose the larger/highly preferred of two possible rewards). In the study by Melotti et al. (2013), pigs needed on average 3.6 sessions (28.8 free choice trials) to significantly choose the lever providing four pieces of apple instead of one. The current study used the same learning criterion (at least seven out of eight free choice trials = success rate of 87.5%), and our results were in the same range (“quality group”−3.2 sessions, 25.6 ± 19.5 free choice trials; “quantity group”−3.9 sessions, 31.2 ± 19.0 free choice trials). In other studies, animals needed a somewhat higher number of trials for successful discrimination, e.g., domestic chickens needed 32 ± 9 to 64 ± 55 trials to discriminate between a rewarded and a non-rewarded object (Croney et al., 2007), and domestic horses took 50.9 ± 10.3 to 74.6 ± 20.4 trials to choose the reward from three patterned cards (Mader and Price, 1980). In those studies, animals had a choice between a reward and no reward, which should have facilitated discrimination learning; however the rewards were not visible and were coded with abstract objects, which complicated their ability to learn the relationship between the object and the reward. In the current study, the piglets from the group with differentially preferred rewards were somewhat (numerically, but not statistically significantly) faster at completing the task (Figure 3). A study in capuchin monkeys with a reversed-reward contingency task showed that learning the task with qualitative differences in reward seemed to be intrinsically easier compared to quantitative differences (Anderson et al., 2008). An additional study in capuchin monkeys showed that contrast in reward quality affected the animals more than contrast in quantity (Talbot et al., 2018). Probably, one of the reasons that affect learning speed and decision making is that qualitative aspects of reward comprise more cues (visual, olfactory) compared to quantitative aspects of reward (just visual cues), which can facilitate discrimination and learning. It should be noted that the piglets in this study were not really hungry due to regular feeding after the experiment, and they did not have completely empty feeding troughs in the morning before testing. Therefore, the reward during the experiment was more a bonus than an essential resource. Furthermore, the contrast in qualitative aspects of reward might result in an increased incentive and motivation compared to the contrast in quantitative aspects (Berridge, 2009).

### Delay maintenance test

In the delay maintenance test, the “quantity group” chose the smaller/least preferred but immediate reward significantly more often, showing less impulse control. This result is supported by the lower maximum delay step of 8 s mastered by two animals of this group compared to the maximum of 24 s achieved by two animals of the “quality group” as well as by a higher number of piglets choosing to wait in the “quality group” (Table 1). Moreover, the “quantity group” completed more sessions than the “quality group,” despite the fact that they did not reach higher delays. This means that they completed more sessions in the single delay steps without reaching the success criterion for the next step or the failure criterion to finish the experiment, i.e., they showed ambivalent behavior. This result indicates that impulse control is partially present but that the opportunity to get a highly preferred reward is more valued by the piglets than the opportunity to get more of a given reward (Hillemann et al., 2014). Additionally, in other species, the importance or incentive value of more preferred rewards has been shown to be higher compared to a higher amount of reward; therefore, the incentive/motivation to wait is higher [corvids: Dufour et al. (2012); Wascher et al. (2012), cockatoos: Auersperg et al. (2013), parrots: Koepke et al. (2015), capuchins: Drapier et al. (2005), dogs: Brucks et al. (2017)] or the discounting of the reward value with increasing delay is lower (Monterosso and Ainslie, 1999). Hillemann et al. (2014) also stated that corvids waited more often when the difference in the preference of the reward was more pronounced (e.g., low-mid vs. low-high). In the current study, rewards which were least preferred were used as contrast to the highly preferred reward, and the results show that the animals were prone to omit any choice with increasing delay, probably due to the low hedonic value of the immediate reward (Berridge, 2009). It has to be taken into account that the piglets (similar to the majority of animals in such tests) were not food-deprived and that they did not incur any nutritional disadvantage by refusing rewards.

With increasing delay, the animals of the “quantity group” tended to switch to the immediate reward while waiting for the larger reward. Melotti et al. (2013) used a delay choice setup with a continuous increase in delay in their study, with no possibility for the pigs to switch once a choice was made and with no visibility of the different amounts of the rewards (i.e., during choice the rewards were represented by the left or right lever, respectively). The results showed that the pigs were able to wait for 12–50 s, tolerating considerably higher delays than were achieved in our study (0–24 s). In contrast to Melotti et al. (2013), our study used a delay maintenance paradigm, giving the pigs the opportunity to switch to the immediate reward at any time, with visible rewards that differed in quantitative aspects (amount) as well as qualitative aspects (preference). With regard to the quantitative aspect of the reward, from reversed reward contingency tasks in primates, it is known that the prepotent impulse to choose a higher over a lower quantity leads to a widespread failure of these animals in this task (Vlamings et al., 2006; Anderson et al., 2008). The same “go for more” effect could be demonstrated in a hybrid delay task showing that monkeys, after initially choosing the larger/later option (delay choice task), were not able to wait until the delivery of the complete reward (delay maintenance/accumulation task; Paglieri et al., 2013). This demonstrates that delay choice and delay maintenance tasks are not equivalent (Addessi et al., 2013). With regard to the visibility aspect of the reward, the performance of primates in the reversed reward contingency task is enhanced when the rewards were covered with colored lids (Vlamings et al., 2006) or were represented by symbols (Addessi and Rossi, 2011). In delay choice tasks, the monkeys more often chose the smaller/sooner option when rewards were represented by symbols or were covered (Genty et al., 2012; Addessi et al., 2014). These results demonstrate that non-visibility of the rewards can override the “go for more” impulse. With regard to the qualitative aspect of the reward, in reversed reward contingency tasks, performance increased when rewards were used that differed in preference (Anderson et al., 2008). In two separate self-control exchange tasks in which animals needed to exchange a less-preferred reward to obtain a more-preferred reward, both chimpanzees (Beran et al., 2016) and capuchin monkeys (Parrish et al., 2018) were more successful on trials with differences in qualitative aspects vs. differences in quantitative aspects of the reward. Finally, in delay-of-gratification tests in pigeons and children, the capacity for impulse control was higher when the rewards were not visible and differed in qualitative aspects (Mischel, 1974; Grosch and Neuringer, 1981). Thus, in the current study, choosing a visible, quantitatively larger (but delayed) reward first (before switching to the smaller immediate reward) rather reflects the initial impulsive choice of “go for more” and not necessarily the intentional choice to wait and therefore may not demonstrate real impulse control in view of a temptation (Addessi et al., 2013; Hillemann et al., 2014). Furthermore, we used a discontinuous increase in delay, i.e., the delay increased only when the individual animal significantly chose to wait, which resulted in a repeated presentation of the same delay. Maybe these effects, i.e., the “go for more” choice with no opportunity of modification and the continuous increase in delay until a stop criterion was reached, led to increased delays for the pigs in the study by Melotti et al. (2013).

According to optimal foraging theory (Charnov, 1976; Stephens and Krebs, 1986; Herrnstein et al., 2000), gain rate (i.e., amount of food/energy intake per unit of time) plays an important role in decision making in inter-temporal choice tasks. Thus, in addition to the quantity of the reward and the delay until delivery of the reward, handling/manipulation time and the duration of inter-trial intervals also matter in choice tasks (Izawa et al., 2003; Aoki et al., 2006; Matsushima et al., 2008). However, we did not control for or manipulate the handling time of the different food amounts, e.g., by making the smaller reward harder accessible (Held et al., 2005). Handling time, regarded as the time the pigs needed to consume the whole amount of food, was difficult to determine exactly. The current setup did not allow us to see when all pieces were consumed, while pigs also tended to further interact with an empty dish. This led to a large inter- as well as intra-individual variability in handling time, independent of the amount of reward. The best strategy for the pigs to maximize profitability, i.e., gain per time (delay + handling), would have been to keep the handling time as short as possible. But the large variability in handling time shows that gain rate maximization seemed not to be the main motivation. Possible factors that contributed to this variability could be intra-individual differences to obtain food, e.g., due to our lack of control on the food intake that each subject had prior to testing, as well as inter-individual differences in foraging behavior (Bolnick et al., 2003). Additionally, our current study design did not allow varying the inter-trial interval, e.g., by trial-specifically adjusting the total trial length. In a recent review, Sjoberg and Johansen (2018) argue that in several studies on delay discounting the delay plays a pivotal role, while the inter-trial interval had little or no effect on choice. Nevertheless, we cannot completely exclude that our results are confounded by other variables like inter-trial interval and handling time. This has to be investigated in further studies specially designed to answer the question on which factors contribute to pigs' choices in delay of gratification tasks.

Compared to several other species, which are able to wait several minutes [primates: Beran et al. (1999), corvids: Hillemann et al. (2014), dogs: Leonardi et al. (2012)] or up to weeks and months [humans: Frederick et al. (2002)], piglets achieved a relatively low level of impulse control, which is also common in several other species [see Figure 13.6. in Stevens and Stephens (2010, p. 380)]. The reasons for these species differences in impulse control, as discussed in Stevens and Stephens (2010), are the structure of the natural habitat (i.e., rich vs. poor) and the domain of selection (i.e., the natural foraging environment including feeding ecology and social competition; Stevens, 2014). Unfortunately, no studies exist that directly investigate the impulse control capacity of wild boar or other wild pig species, especially in terms of economic decision making. Only one study describes a coincidental but locally restricted observation of wild boar washing soiled food in a zoo (Sommer et al., 2016). The authors argued that the capacity of delaying gratification could have facilitated this behavior. However, Korte et al. (2009) presented a framework discussing the effects of artificial selection of production traits in farm animals with regards to their metabolism, hormone and brain physiology and, consequently, their behavior. The comparably low impulse control of piglets could reflect the “hawk behavioral strategy,” which, accompanied by active, aggressive and bold behavior, increases vulnerability to injurious behavior and stereotypes, such as tail biting. Despite this, and similar to other non-domesticated species, pigs showed considerable individual differences in their impulse control capacity. Two factors that could contribute to this high inter-individual variation are diverging social behavior and personality traits. For example, within the scope of “social foraging theory,” individuals can adapt different foraging strategies (e.g., producer, scrounger) and therefore could show differences in impulse control depending on their position within the social network (Giraldeau and Dubois, 2008). Moreover, individuals possess different personalities that are shaped by evolution and ecology (Dingemanse and Wolf, 2010; Réale et al., 2010). For example, within the scope of “coping theory” individuals can adapt to be more active or passive in the face of challenges and thus may also show differences in impulse control (Lazarus, 1993; Zebunke et al., 2015, 2017). This is a starting point for further studies investigating the relationships between impulse control and personality as well as social behavior traits. Moreover, especially in light of field applications, individual differences in impulse control could also play a role in the problematic behaviors that emerge in intensive housing conditions due to stressful conditions, such as injurious behavior (e.g., aggression, tail biting) and stereotypes. Further knowledge about the features, mechanisms and relationships of impulse control to other traits could help to find approaches that increase farm animal welfare.

## Conclusion

The results of the current study show that in studies that rely on the motivation of the participating subjects, care must be taken in reward selection, which makes a preliminary preference test useful. Similar to other species, pigs showed higher impulse control when the reward differed in qualitative aspects rather than quantitative aspects. The inter-individual variance in impulse control measured in this study could be seen as a starting point for future studies aimed at the understanding of the relevance of impulse control in farmed species in the context of animal welfare. Broader knowledge regarding impulse control could help to assess the reaction of the animals to differentially available resources and to adapt husbandry practices to species/breed specific demands and individual differences in this behavioral aspect.

## Author contributions

MZ conceived and planned the experiments, performed the analytic calculations and wrote the manuscript. MK carried out the experiments and collected the data. NM helped shape the analysis and manuscript. JL and BP contributed to the idea, the planning and supervised the project.

### Conflict of interest statement

The authors declare that the research was conducted in the absence of any commercial or financial relationships that could be construed as a potential conflict of interest.

## Supplementary material

Supplementary Table 1 Overview of the omissions during the preference test: percentage of omissions with regard to the number of total trials (N = 45) per animal, percentage of items refused by the individual animals (group = “quantity” [amount: 1:4], “quality” [differentially preferred items: low:high]; animal = 1-10) from both groups (group = “quantity” [amount: 1:4], “quality” [differentially preferred items: low : high]; animal = all) as well as in total (group = all, animal = all). Averaged values within the groups and across both groups are highlighted in bold.

Supplementary Table 2 Overview of the choices during the preference test: percentage of choices made with regard to the number of total trials (N = 45) per animal, percentage of items chosen by individual animals (group = “quantity” [amount: 1:4], “quality” [differentially preferred items: low:high]; animal = 1-10) from both groups (group = “quantity” [amount: 1:4], “quality” [differentially preferred items: low:high]; animal = all) as well as in total (group = all, animal = all). Averaged values within the groups and across both groups are highlighted in bold. Individual, highly preferred items are highlighted in bold and italicized. The food items chosen as individual rewards for the following tests are underlined. For the group with qualitative differences in reward (“group quality,” differentially preferred items: low:high), the individual food items used as the least preferred reward are marked as crossed-out characters.

Supplementary Table 3 Overview of the performance of each pig during the discrimination test over the course of the sessions for both groups (“quantity”: with a quantitative difference in reward, i.e., 1 vs. 4 pieces of the most preferred reward; “quality”: with a qualitative difference in reward, i.e., 1 piece of the most preferred vs. 1 piece of the least preferred reward). Each symbol indicates a decision during a trial: black circle = choice of the larger/highly preferred reward; white circle = choice of the smaller/least preferred reward; - = omission.

Supplementary Table 4 Overview of the performance of single pigs in the two groups (“quantity”: with a quantitative difference in reward, i.e., 1 vs. 4 pieces of the most preferred reward; “quality”: with a qualitative difference in reward, i.e., 1 piece of the most preferred vs. 1 piece of the least preferred reward) during the course of the delay maintenance test with increasing delays (2 s, 4 s, 8 s, 16 s, 24 s, 32 s) to the larger/highly preferred reward. Each symbol indicates a decision during a trial: black circle = successful waiting for the larger/highly preferred reward; ⋂ = switching, i.e., first choosing to wait, but switching to the immediate reward before the end of the delay; white circle = choosing the immediate smaller/least preferred reward; - = omission. The rows within each individual indicate repeated sessions within one delay step due to not reaching the learning criterion (significant binomial testing of successful against unsuccessful waiting, i.e., switching, immediate, omission).

Supplementary Video 1 Video sample of a pig of the “quantity group” (amount: left = 1 piece vs. right = 4 pieces) choosing to wait a delay of 8 s for the larger reward (right side).

Supplementary Video 2 Video sample of a pig of the “quantity group” (amount: left = 1 piece vs. right = 4 pieces) choosing the small, immediate reward (left side).

Supplementary Video 3 Video sample of a pig of the “quantity group” (amount: left = 1 piece vs. right = 4 pieces) switching from the larger (right side) to the smaller reward (left side) during the delay of 8 s.
