# Supplementary material for: Better, Not Just More—Contrast in Qualitative Aspects of Reward Facilitates Impulse Control in Pigs
Source: Front Psychol. 2018 Nov 6;9:2099. doi: 10.3389/fpsyg.2018.02099 (PMC6232270; doi:10.3389/fpsyg.2018.02099)
Supplement: Supplementary Table 1 — Overview of the omissions during the preference test: percentage of omissions with regard to the number of total trials (N = 45) per animal, percentage of items refused by the individual animals (group = “quantity” [amount: 1:4], “quality” [differentially preferred items: low:high]; animal = 1-10) from both groups (group = “quantity” [amount: 1:4], “quality” [differentially preferred items: low : high]; animal = all) as well as in total (group = all, animal = all). Averaged values within the groups and across both groups are highlighted in bold. [file Table_1.docx]

| group | animal | %omissions/TotalTrials | %pellets | %penne | %M&M`s^®^ | %apple | %cheese | %sausage |
| --- | --- | --- | --- | --- | --- | --- | --- | --- |
| quantity | 1 | 0.0 |  |  |  |  |  |  |
|  | 2 | 6.7 | 33.3 | 33.3 | 33.3 | 0.0 | 0.0 | 0.0 |
|  | 3 | 11.1 | 30.0 | 20.0 | 30.0 | 0.0 | 10.0 | 10.0 |
|  | 4 | 20.0 | 33.3 | 33.3 | 33.3 | 0.0 | 0.0 | 0.0 |
|  | 5 | 0.0 |  |  |  |  |  |  |
|  | 6 | 0.0 |  |  |  |  |  |  |
|  | 7 | 0.0 |  |  |  |  |  |  |
|  | 8 | 20.0 | 33.3 | 33.3 | 33.3 | 0.0 | 0.0 | 0.0 |
|  | 9 | 2.2 | 0.0 | 50.0 | 50.0 | 0.0 | 0.0 | 0.0 |
|  | 10 | 2.2 | 50.0 | 0.0 | 50.0 | 0.0 | 0.0 | 0.0 |
|  | **all** | **6.2** | **32.1** | **30.4** | **33.9** | **0.0** | **1.8** | **1.8** |
| quality | 1 | 24.4 | 31.8 | 22.7 | 22.7 | 22.7 | 0.0 | 0.0 |
|  | 2 | 2.2 | 50.0 | 50.0 | 0.0 | 0.0 | 0.0 | 0.0 |
|  | 3 | 8.9 | 50.0 | 25.0 | 25.0 | 0.0 | 0.0 | 0.0 |
|  | 4 | 20.0 | 33.3 | 33.3 | 33.3 | 0.0 | 0.0 | 0.0 |
|  | 5 | 31.1 | 32.1 | 21.4 | 25.0 | 7.1 | 7.1 | 7.1 |
|  | 6 | 28.9 | 30.8 | 19.2 | 23.1 | 7.7 | 11.5 | 7.7 |
|  | 7 | 13.3 | 33.3 | 33.3 | 33.3 | 0.0 | 0.0 | 0.0 |
|  | 8 | 15.6 | 28.6 | 35.7 | 28.6 | 0.0 | 7.1 | 0.0 |
|  | 9 | 4.4 | 50.0 | 50.0 | 0.0 | 0.0 | 0.0 | 0.0 |
|  | 10 | 2.2 | 50.0 | 50.0 | 0.0 | 0.0 | 0.0 | 0.0 |
|  | **all** | **15.1** | **33.8** | **27.2** | **25.0** | **6.6** | **4.4** | **2.9** |
| **in total** | | **10.7** | **33.3** | **28.1** | **27.6** | **4.7** | **3.6** | **2.6** |
