# Supplementary material for: Better, Not Just More—Contrast in Qualitative Aspects of Reward Facilitates Impulse Control in Pigs
Source: Front Psychol. 2018 Nov 6;9:2099. doi: 10.3389/fpsyg.2018.02099 (PMC6232270; doi:10.3389/fpsyg.2018.02099)
Supplement: Supplementary Table 2 — Overview of the choices during the preference test: percentage of choices made with regard to the number of total trials (N = 45) per animal, percentage of items chosen by individual animals (group = “quantity” [amount: 1:4], “quality” [differentially preferred items: low:high]; animal = 1-10) from both groups (group = “quantity” [amount: 1:4], “quality” [differentially preferred items: low:high]; animal = all) as well as in total (group = all, animal = all). Averaged values within the groups and across both groups are highlighted in bold. Individual, highly preferred items are highlighted in bold and italicized. The food items chosen as individual rewards for the following tests are underlined. For the group with qualitative differences in reward (“group quality,” differentially preferred items: low:high), the individual food items used as the least preferred reward are marked as crossed-out characters. [file Table_2.docx]

| group | animal | %choices/TotalTrials | %pellets | %penne | %M&M`s^®^ | %apple | %cheese | %sausage |
| --- | --- | --- | --- | --- | --- | --- | --- | --- |
| quantity | 1 | 100.0 | 15.6 | 6.7 | ***22.2*** | 15.6 | 20.0 | 20.0 |
|  | 2 | 93.3 | 2.4 | 2.4 | 11.9 | 23.8 | ***35.7*** | 23.8 |
|  | 3 | 88.9 | 0.0 | 2.5 | 17.5 | ***35.0*** | 25.0 | 20.0 |
|  | 4 | 80.0 | 0.0 | 0.0 | 0.0 | ***33.3*** | ***33.3*** | ***33.3*** |
|  | 5 | 100.0 | 6.7 | ***24.4*** | 6.7 | 17.8 | ***24.4*** | 20.0 |
|  | 6 | 100.0 | 15.6 | 17.8 | 4.4 | 20.0 | 20.0 | ***22.2*** |
|  | 7 | 100.0 | 6.7 | 20.0 | ***22.2*** | 20.0 | 17.8 | 13.3 |
|  | 8 | 80.0 | 0.0 | 0.0 | 2.8 | 33.3 | 27.8 | ***36.1*** |
|  | 9 | 97.8 | 6.8 | 4.5 | 13.6 | ***29.5*** | 20.5 | 25.0 |
|  | 10 | 97.8 | 13.6 | 13.6 | 11.4 | 20.5 | 18.2 | ***22.7*** |
|  | **all** | **93.8** | **7.1** | **9.7** | **11.6** | **24.4** | **23.9** | **23.2** |
| quality | 1 | 75.6 | 0.0 | 5.9 | ~~2.9~~ | 14.7 | 32.4 | ***44.1*** |
|  | 2 | 97.8 | 0.0 | ~~4.5~~ | 20.5 | ***29.5*** | 25.0 | 20.5 |
|  | 3 | 91.1 | 2.4 | 9.8 | ~~2.4~~ | 24.4 | 26.8 | ***34.1*** |
|  | 4 | 80.0 | 0.0 | ~~0.0~~ | 0.0 | 30.6 | ***36.1*** | 33.3 |
|  | 5 | 68.9 | 0.0 | ~~0.0~~ | 3.2 | 29.0 | 32.3 | ***35.5*** |
|  | 6 | 71.1 | 0.0 | ~~0.0~~ | 3.1 | ***34.4*** | ***34.4*** | 28.1 |
|  | 7 | 86.7 | 5.1 | 7.7 | ~~5.1~~ | ***30.8*** | 25.6 | 25.6 |
|  | 8 | 84.4 | 0.0 | 5.3 | ~~2.6~~ | 28.9 | ***34.2*** | 28.9 |
|  | 9 | 95.6 | 2.3 | ~~4.7~~ | ***25.6*** | 23.3 | 23.3 | 20.9 |
|  | 10 | 97.8 | 6.8 | ~~0.0~~ | 15.9 | 22.7 | ***29.5*** | 25.0 |
|  | **all** | **84.9** | **1.8** | **3.9** | **8.9** | **26.7** | **29.6** | **29.1** |
| **in total** | | **89.3** | **4.6** | **7.0** | **10.3** | **25.5** | **26.6** | **26.0** |
